# Blood pressure trajectory modeling in childhood: birth-cohort study

**DOI:** 10.1186/s40885-019-0133-9

**Published:** 2020-01-15

**Authors:** Jung Won Lee, Nameun Kim, Bohyun Park, Hyesook Park, Hae Soon Kim

**Affiliations:** 1 Department of Pediatrics, Ewha Womans University College of Medicine, 25, Magokdong-ro 2-gil, Ganseo-gu, Seoul, 07804 South Korea; 2 Department of Preventive Medicine, Ewha Womans University College of Medicine, 25, Magokdong-ro 2-gil, Ganseo-gu, Seoul, 07804 South Korea

**Keywords:** Blood pressure, Children, Trajectory

## Abstract

**Background:**

Systolic blood pressure (SBP) and diastolic blood pressure (DBP) tends to increase with age and increase in proportion to body weight and height. Recent epidemiological and longitudinal cohort studies have found that high BP in children can be progressed into hypertension (HTN) in adulthood. Therefore, the aim of this study is that we monitor and analyze the tendency of the BP trajectory in children from the age of 3 years to the age of 10 years.

**Method:**

A total of 767 subjects were gathered from Ewha Birth and Growth cohort study. We observed and analyzed the data of 65 subjects which were completely repeated measures for 6 times as 3, 5, 7, 8, 9, 10 years old follow-up. We collected retrospective information such as BP and anthropometric data measured for children and constructed the trajectory models of SBP and DBP in early stage of life.

**Results:**

Three distinct trajectories on SBP and DBP from 3 to 10 years old were identified. As a result of SBP, 82.7% (*n* = 54) of subjects experienced moderate SBP levels maintained stable levels; 13.7% (*n* = 9) of subjects experienced a rapid increase as the age increase; 3.6% (*n* = 2) of subjects experienced high SBP levels throughout follow-up as moderate grade. For DBP, 6.7% (*n* = 4) of subjects started with low levels and experienced generally a gradual grade; 61.7% (*n* = 41) of subjects started with moderate levels and experienced a steep increase at 7-years-old; 31.6% (*n* = 20) of subjects experienced a rapid increase on DBP levels.

**Conclusion:**

The result of study shows tendency of increase BP as the age increase. This research inspires that we verify risk group and risk factor in early stage of life with trajectory modeling for the HTN prevention in adulthood.

## Background

The obesity epidemic in children makes it plausible that prevalence rates of elevated blood pressure (BP) are increasing over time [1]. In national surveys from the 1960s to the 1990s, the prevalence of overweight in children grew from 5 to 11% [2]. Current estimates suggest that approximately 18.5% of children 2–19 years of age are obese [3]. The prevalence of pediatric hypertension (HTN) in children has ranged from 2 to 4% based on previous guidelines [4]. High BP is consistently grater in boys [15–19%] than in girls [7–12%]. Primary HTN in children has become increasingly common in association with obesity and obese children are at approximately a 3-fold higher risk for HTN than non-obese children [5].

Therefore, pediatric HTN has undergone an epidemiological shift. The conventional wisdom has been that HTN in children is a relatively rare condition most commonly associated with renal disease. In actuality, secondary HTN in children resulting from renal disease has become far less common than that related to primary (ie, essential) HTN.

It has been recognized for a long time that elevated BP in the young may persist and progress into adult HTN [6, 7]. Longitudinal cohort studies also revealed the predicted BP trajectory lines, starting from childhood, and cardiovascular (CV) risks in adulthood [8–10].

With extended life expectancy and improved quality of life, more strictly controlled BP in childhood with HTN is recommended. Recent research among older adults suggests there may be subgroups with different BP trajectories. Identifying subgroups at risk of developing adult HTN early in life can inform effective risk reduction efforts [10].

The American Academy of Pediatrics (AAP) has endorsed NHLBI guidelines that recommended all children over 3 years of age have BP measured at least annually for early recognition of BP abnormalities such as hypertension or syncope [11].

However, it is also highly demanding process to effect the cooperation of very young children during BP measurement for reliable measured data due to higher incidence of white coat HTN compared with adults [12] and there is no standardized normal BP reference value and percentile of preschool children in Korea.

Therefore, the purpose of our study were to (1) monitor and analyze the tendency of the BP in children in Korea, (2) identify the trend of change of BP by time, and (3) identify the risk group for BP level through trajectory modeling.

## Methods

### Study subjects

This study was conducted as part of the Ewha Birth and Growth Cohort study, which was established at Mokdong Hospital, Ewha Womans University from 2001 to 2006. We enrolled women who visited to prenatal care between 24 and 28 weeks of gestation and their children (*n* = 940). The first follow-up survey was conducted when the children were 3 years of age from November 2005 to July 2010 (*n* = 472). The follow-up survey was carried out at 3, 5, 7 to 10 years of age or older, and 10 year follow up was completed. Details of this birth cohort were reported previously [13, 14].

This study included 65 subjects who participated in all 3, 5, 7 to 10 years old follow-up examinations. Data were collected from November 2005 to May 2018. Those who participated in the follow up survey underwent anthropometric measurements, completed questionnaires, and provided blood and urine samples. The study protocol was approved by the Institutional Review Board on Human Subjects at Ewha Womans University Hospital. Written informed consent for participation in the study was obtained from all parents or guardians and participating children when the subjects visited the hospital for check-up survey. All processes were performed in accordance with relevant guidelines and regulations.

### Anthropometric and blood pressure measurements

All the follow up visit, well trained researchers measured current anthropometric and blood pressure. Heights and weights without shoes and with light clothing were measured to the nearest 0.1 cm or 0.1 kg using a stadiometer and calibrated scale, respectively (DS-102, Dong Sahn Jenix, Seoul, Korea). Body mass index (kg/m^2^) was calculated as weight divided by height squared. Blood pressure was measured twice after the subjects had been relaxed for 5 min using an automatic blood pressure (BP) monitor (Dinamap Procure 200, GE, Milwaukee, WI) with an appropriate size of BP cuff of the upper left arm. The average of the two measurements was used for statistical analysis. The Mean Arterial Pressure (MAP) was calculated as DBP + (SBP-DBP)/3.

### Statistical analysis

The data were analyzed using SAS version 9.4 software package (SAS Institutes, Cary, NC, USA). Continuous data are expressed as the mean ± standard deviation and categorical data are expressed as the number of subjects with percentages.

Trajectory analysis (SAS Proc Traj) was used to determine clusters of participants who followed similar long-term patterns of SBP, DBP and MAP measured 6 times repeatedly [15]. Because the number of subjects was relatively small, the number of trajectory groups was limited from two to three categories. We used the Bayesian information criterion to evaluate the model fit.

## Results

### General characteristics of study subjects

Sixty five subjects were enrolled in our birth cohort. Of the 65 subjects studied, 35 were boy (53.8%) and 30 were girl (46.2%) which showed a relatively similar distribution. The BMI at 3 years old was 15.3 kg/m^2^ and showed a tendency to increase with age reaching 18.0 kg/m^2^ at age 10.

SBP, DBP and MAP tended to increase with age, which was similar for both boys and girls. After age 9, the increase in systolic blood pressure slowed. (Table 1).

**Table 1:**
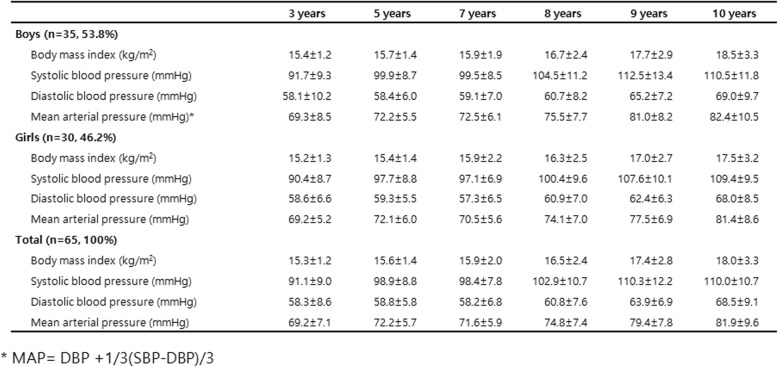
Characteristics of the study participants

### Trajectory modeling in preschool-prepuberty-puberty follow up

**Fig. 1:**
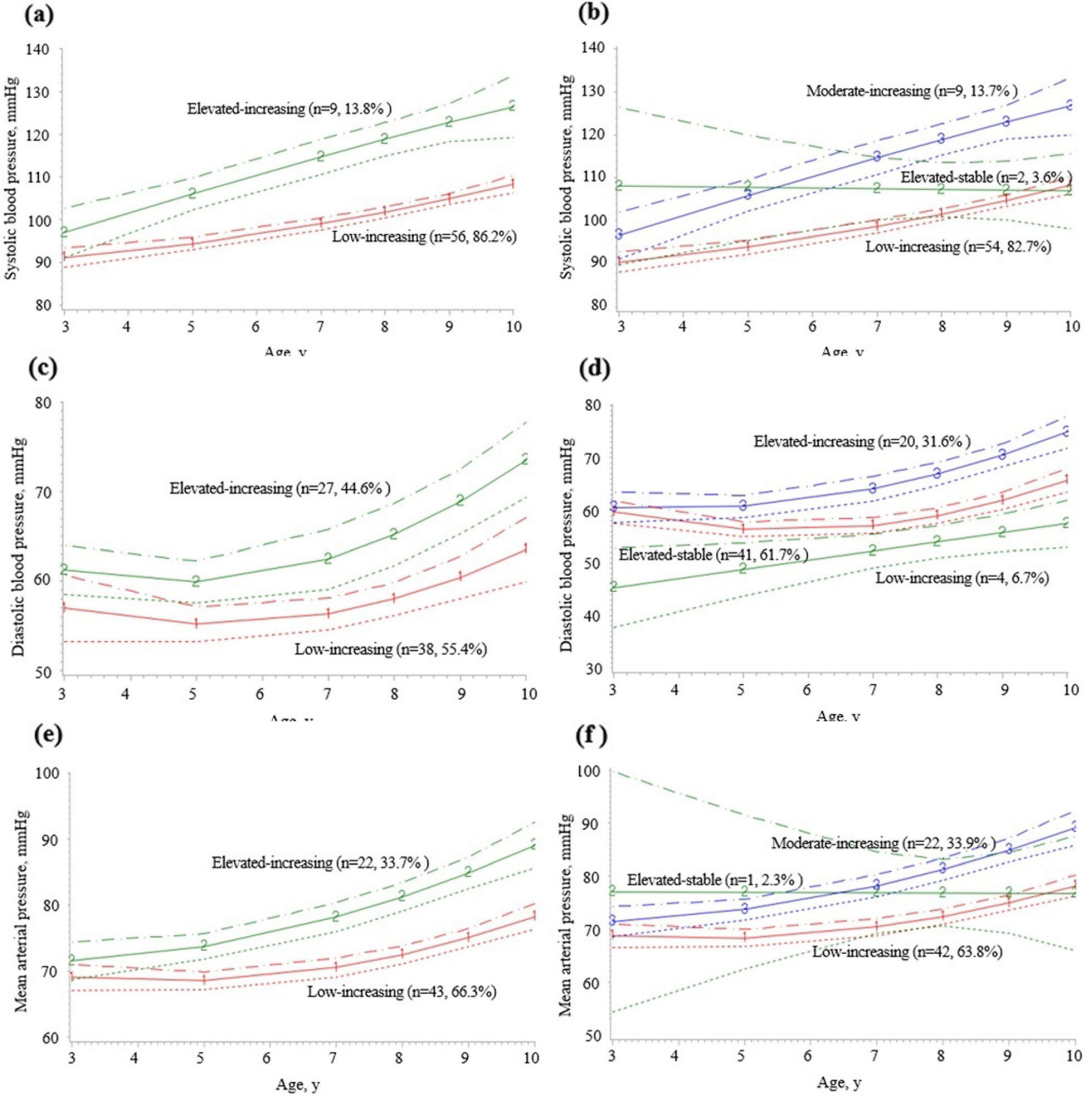
Blood pressure trajectories in early childhood. **a** Two discrete SBP trajectories. **b** Three discrete SBP trajectories. **c** Two discrete DBP trajectories. **d** Three discrete DBP trajectories. **e** Two discrete MAP trajectories. **f** Three discrete MAP trajectories

Using the trajectory analysis program, two or three life course discrete BP trajectories in children were identified (Fig. 1).

In two discrete SBP trajectories, 86.2% (95% CI: 77.8–94.6, *n* = 56) of subjects started with low levels and experienced a mild increase in BP throughout follow-up (low-increasing group); and 13.8% (95% CI: 5.4–22.2, *n* = 9) had started with high BP levels with more rapid increase (elevated-increasing group) (Fig. 1-a).

In three discrete SBP trajectories, 82.7% (95% CI: 73.5–91.9, *n* = 54) had low-increasing group, 13.7% (95% CI: 5.3–22.1, *n* = 9) started with moderate levels and experienced rapid increase in BP (moderate increasing group), and two subjects (3.6%) had relatively elevated BP levels throughout (elevated-stable group) (Fig. 1-b).

In two discrete DBP trajectories, 55.4% (95% CI: 43.3–67.5, *n* = 38) of subjects started with low levels and experienced a mild increase in BP (low-increasing group); 44.6% (95% CI: 32.5–56.7, *n* = 27) had high BP levels (elevated-increasing group) (Fig. 1-c). Both groups maintained stable blood pressure levels from the age of 3 to 5 years and have shown an increasing pattern since the age of 5. The increase in BP from 5 to 7 years was moderate. After age 7, the level of DBP increase in both groups was significant. Even in three discrete DBP trajectories, it tended to be similar to that of two discrete DBP trajectories. (Fig. 1-d).

In two discrete MAP trajectories, 66.3% (95% CI: 54.8–77.8, *n* = 43) of subjects started with low levels and experienced a mild increase in MAP throughout follow-up (low-increasing group); and 33.7% (95% CI: 22.2–45.2, *n* = 22) started with high BP levels with mild increase (elevated-increasing group). Both group showed steep increase in MAP after 7 years old (Fig. 1-e).

In three discrete MAP trajectories, both low increasing group [63.8% (95% CI: 52.1–75.5, *n* = 42)] and moderate increasing groups [33.9% (95% CI: 22.4–45.4, *n* = 22)] showed slow increase of MAP between the age of 3 and 7. However, MAP trajectory showed rapid increase MAP after 7 years old. Interestingly, MAP showed steady and minimal increase from 3 to 10 years in elevated stable group (Fig. 1-f). It must be due to a result of 1 subject (2.3%) and the subject number needs to be expanded.

High MAP group at age 3 showed large increase in BP over time than low MAP group. The difference between the high and low MAP groups was estimated to be 2.1 mmHg at age 3 and about 12.2 mmHg at age 10 which is much larger. So it is thought to be important to maintain a proper level of blood pressure from infancy.

We devided into four groups such as, (1) elevated-increasing both SBP and DBP (group 1), (2) elevated-increasing SBP only (group 2), (3) elevated-increasing DBP only (group 3), (4) low-increasing both SBP and DBP (group 4). Both systolic and diastolic BP increasing group (group 1) had 12.3% of subjects. It is necessary to close follow-up BPs of high risk group until adulthood period eventhough absolute number is small (*n* = 8).

We analyzed the relationship between birth weight, weight gain, and maternal HTN according to SBP/DBP and MAP trajectory. Mean birth weight in group 1 was higher (3470 g) than mean total group (2987 g) (*P* = 0.15) and change in weight until 3 years in elevated-increasing SBP only group (group 2) was most among other groups. Birth weight in elevated-increasing MAP group was higher than low-increasing MAP group without statistical significance (*P* = 0.23). There was no correlation between maternal hypertension and SBP/DBP trajectory (*P* = 0.89). MAP trajectory (*P* = 0.29).

## Discussion

In this study, we used a repeated blood pressure data from the age of 3 to 10 years old and conducted a trajectory model in which the blood pressure level moves with a similar tendency over time. Trajectory model describes the course of a measured variables over age or time, identifies groups of individuals following similar progressions of some phenomenon over time and estimates the effects of covariates on trajectory shape.

Among the reports insisting that childhood HTN persists over time into adulthood, studies on this perspective using the Bogalusa heart study for nearly about 40 years are well known [16]. About 10 years later, Bao et al. revealed that among 1505 individuals, the subjects who were in the highest quintile of their BP during young children aged 5 to 14 were much more likely to develop HTN 15 years later by tracking of elevated BP from childhood to adulthood. They also demonstrated that the weight gain from childhood to adulthood was an independent predictor of HTN [6]. Our study showed that weight gain until 3 years in elevated-increasing SBP only group was most among other groups and birth weight in elevated-increasing both SBP and DBP group was higher. However, subject number was relatively small in this study. Therefore, there was no statistical significance correlation between birth weight, weight gain and BP trajectories. So we think it is necessary to further increase subject number, and expect to find further robust correlation.

Forty-five percent of adults with high systolic blood pressure are known to have blood pressure of more than 90 percentile in childhood, while children with blood pressure of more than 90 percentile are 2.4 times more likely to develop adult hypertension than children with lower blood pressure [17].

Webber et al. reported that great persistent tracking for CV risk factors was noted for height and weight in 2236 children over a 5-year period. For systolic BP, they reported that more than 30% of children initially high also remained high in the second examination [18]. Staley et al. conducted interesting study on association between maternal high BP and offspring’s trajectory changes during childhood were examined. The differences in BP between offspring of hypertensive and normotensive pregnancies remain consistent across childhood and adolescence according to higher maternal BP in early pregnancy, rather than by pregnancy-related BP changes [19].

There already have been innumerable reports highlighting the CV risks of HTN in youth. Among target organ damage due to HTN, left ventricular hypertrophy (LVH), vascular stiffness, and increased carotid-intima thickness are considered as surrogate markers.

A large longitudinal study showed longitudinal BP trajectories from childhood and the impact of level-independent childhood BP trajectories on adult LVH and remodeling patterns implying the impact of BP trajectories on adult LVH originate in childhood and adolescence is a crucial window for early prevention of LVH in later life [20].

However, the prevalence rate of high blood pressure in children is reported differently from each other due to different blood pressure measuring method and sphygmomanometer as well as the range of children can be very large and the younger children can have higher incidence of white coat hypertension, which makes the diagnosis different [21]. There are several reports on the inaccuracy in pediatric outpatient BP measurement [22].

Blood pressure measurement with mercury sphygmomanometer is gold standard. But, it is difficult to apply to infants and young children because they are not able to keep steady and to take korotkoff sound. Oscillometric blood pressure device are widely used instead. Because it is easy to use, can minimize the measurement error, measure average arterial blood pressure, followed by systolic and diastolic blood pressure, provides a significant approximation to actual BP [6]. The AAP practice guideline for pediatric HTN (2017) provides extensive areas for the recognition and identification of pediatric patients with HTN and include new thresholds for pediatric HTN based on percentile references calculated from healthy weighted pediatric population [23].

The significance of our study is that it is first orbital model of BP in early childhood period. This may contribute to researches on pediatric population belongings to the health disadvantaged class. It could also be used as a basic data for the development of high blood pressure prevention policy and program.

There are some limitations of this study. First, although we were able to examine the BP trajectory model of early children between 3 and 10 years in Korean population for the first time, the final number of analyzed was small. Second, although we were able to examine the effect of BMI on BP, individual height and weight data were restricted to compare which component further affects on BMI.

## Conclusion

In conclusion, SBP, DBP and MAP showed tendency of increase BP as the age increase. This research inspires that we verify risk group and risk factor in early stage of life with trajectory modeling for the HTN prevention in adulthood. In the future, further study to identify standard guidelines for BP control before school age are needed and it is believed to be important to maintain an appropriate level of blood pressure from infancy.

## Data Availability

Not applicable.

## Abbreviations

AAP: American Academy of Pediatrics
BP: Blood pressure
CV: Cardiovascular
DBP: Diastolic blood pressure
HTN: Hypertension
MAP: Mean arterial pressure
SBP: Systolic blood pressure
